# Entry of the antipsychotic drug, olanzapine, into the developing rat brain in mono- and combination therapies

**DOI:** 10.12688/f1000research.128074.1

**Published:** 2022-12-02

**Authors:** Yifan Huang, Fiona Qiu, Mark Habgood, Shuai Nie, Katarzyna Dziegielewska, Norman Saunders

**Affiliations:** 1 Department of Neuroscience, Monash University, Melbourne, Victoria, 3004, Australia; 2 Department of Biochemistry and Pharmacology, University of Melbourne, Parkville, Victoria, 3010, Australia; 3 Melbourne Mass Spectrometry and Proteomics Facility, Bio 21 Molecular Science and Biotechnology Institute, University of Melbourne, Parkville, Victoria, 3010, Australia

**Keywords:** blood brain barrier, placental barrier, P-glycoprotein, cerebrospinal fluid, choroid plexus, drug transfer, olanzapine, drug interaction

## Abstract

**Background:** Olanzapine is used to treat schizophrenia and bipolar disorder in women of childbearing age. Continuation of psychotropic medications throughout pregnancy and lactation is often required as cessation could be dangerous for both mother and child. However, there is a lack of information on the transfer of these drugs into the developing brain.

**Methods:** Sprague Dawley rats at three developmental ages: embryonic day E19, postnatal day P4 and non-pregnant adult females were administered unlabelled or radiolabelled ( ^3^H) olanzapine (0.15 mg/kg) either as monotherapy or in combination with each of seven other common medications. Similar injections were administered to pregnant E19 females to investigate placental transfer. Olanzapine in plasma, cerebrospinal fluid (CSF) and brain was measured by liquid scintillation counting after a single dose (acute) or following 5 days of treatment (prolonged).

**Results:** Olanzapine entry into brain and CSF was not age-dependent. Prolonged olanzapine treatment reduced placental transfer from 53% to 46% (p<0.05). Co-administration of digoxin or lamotrigine with olanzapine increased its entry into the fetal brain, whereas paracetamol decreased its entry into the CSF. Placental transfer of olanzapine was increased by co-treatment with cimetidine and digoxin, whereas co-treatment with lamotrigine, paracetamol or valproate led to a substantial decrease. Repeated co-treatment of digoxin and olanzapine increased olanzapine transfer into the brain and CSF, but not across the placenta. Overall entry of olanzapine from maternally administered drugs into the fetal brain was higher after combination therapy with cimetidine and digoxin.

**Conclusions:** Co-administration of olanzapine with some commonly used drugs affected its entry into the fetus and its developing brain to a greater extent than in adults. It appears that protection of the fetal brain for these drugs primarily comes from the placenta rather than from the fetal brain barriers. Results suggest that drug combinations should be used with caution particularly during pregnancy.

## Abbreviations

ABC: ATP-binding cassette

BCRP: breast cancer resistance protein

CSF: cerebrospinal fluid

MRP: multidrug resistance protein

Pgp: P-glycoprotein

SLC: solute carrier

## Introduction

Psychological and neurological problems are becoming more prevalent and require a concerted approach to develop more effective and safe treatment regimes. During pregnancy, women are hesitant to take medications for fear of harming their unborn child. Clinicians have the difficult task of weighing up maternal benefit provided by the treatment versus potential risks to the developing baby. This is complicated by limited knowledge of drug safety due to lack of clinical trials conducted in pregnant women for obvious ethical reasons. The evidence base available to clinicians and their patients is limited to clinical experience, expert opinion, data bases (e.g. The Royal Women’s Hospital, Melbourne) and guidance from up-to date reports on child and maternal outcomes of women prescribed drugs during pregnancy ( Briggs *et al.*, 2021). Recently the inclusion of pregnant women in clinical trials has been advocated for newly developed medications ( van der Graaf *et al.*, 2018; Stock and Norman, 2019) but is unlikely to occur for established treatments because of the cost involved. For current therapies the risk remains as cessation of treatment during pregnancy could be harmful for both mother and child.

For anti-psychotic drugs such as olanzapine, many women are recommended to continue taking their medication during pregnancy as maternal benefits are deemed to outweigh any potential fetal risks. A review of olanzapine exposure during the first trimester of pregnancy revealed a congenital malformation rate of 3.5%, which is the same as the background malformation rate in the general population ( Ennis and Damkier, 2015); this indicates that olanzapine does not appear to increase the risk of congenital malformations. However, there are potential neurological problems that need to be considered. It is not known to what degree olanzapine is able to cross the placenta and enter the fetal brain although a few studies have investigated placental transfer in *ex vivo* ( Schenker *et al.*, 1999) and *in vivo* ( Newport *et al.*, 2007) in human material.

In the present study we investigated the entry of olanzapine from the mother’s blood across the placenta and into the late gestation fetal brain and into the brain of postnatal rats exposed to the drug acutely and over several days. Effects of co-administration of several commonly used therapeutics (digoxin, cimetidine, lamotrigine, fluvoxamine, lithium, paracetamol, valproate) on olanzapine transfer were also investigated. Results showed that prolonged exposure to olanzapine affected its brain entry in postnatal but not fetal animals and co-administration of several of the drugs tested significantly affected the placental transfer and fetal brain entry. These results are discussed in the context of potential application to clinical care.

## Methods

### Ethical statement

All animal experimentation was approved by the University of Melbourne Animal Ethics Committee (Ethics Approval AEC: 10270 approved 30.12.2019) and conducted in compliance with Australian National Health and Medical Research Guidelines. All animals were assessed as healthy prior to commencement of experiments. All surgeries were short term and conducted under terminal anaesthesia. Animals were handled only by experienced researchers in such a way as to minimise their stress and every effort was made to ameliorate any suffering. For animals treated over several days they were monitored before and after every treatment ensuring that there were no abnormalities in weight (>15%), appearance (fur, wounds) or behaviour (vocalisation, respiration, movements). All aspects of the study conformed to the ARRIVE guidelines ( Huang *et al.*, 2022)

### Animals

The Sprague–Dawley ( RRID: RGD_728193) strain of *Rattus norvegicus* was used in this study supplied by the University of Melbourne Biological Research Facility. Animals were housed in groups of 2–4 (adults) or full litters per cage (25cm x 35cm x 25cm on Breeders Choice paper bedding, made from 99% recycled paper and biodegradable with no added chemicals), on a 12h light/dark cycle with *ad libitum* access to food (dry pellets of a fixed formulation for rats (Speciality Feeds, Western Australia) and water.

The ages studied were: fetuses from time-mated females (all primigravida) at embryonic day (E)19, pups at postnatal day (P)4 and non-pregnant adults. Dating of animals was based on taking E0 as the day a vaginal plug was identified and P0 as the day of birth. E19 was chosen as it is a fetal stage of development where adequate volumes of blood and cerebrospinal fluid (CSF) can be obtained for analysis without pooling ( Dziegielewska *et al.*, 1981), and individual pups can be injected intraperitoneally ( *i.p.*) while still inside the uterine horn and kept viable for periods of time ( Koehn *et al.*, 2019; Koehn *et al.*, 2020). P4 was chosen because its stage of brain development is similar to that of very premature but viable human infants of 22–24 weeks gestation ( Clancy *et al.*, 2001; Workman *et al.*, 2013). Additionally, the results in this paper can be compared with data from previous studies using other drugs at these ages ( Koehn *et al.*, 2019; Koehn *et al.*, 2020; Toll *et al.*, 2021). Animal numbers ( Table 1) were based on previous experience of such experiments and were the minimum number required to detect a significant difference between groups at p<0.05. Animals were selected for treatment groups to ensure weights were statistically similar between groups that were being compared. Animals were allocated to experiments by the Animal House staff, who had no knowledge of the particular experiments to be performed; experimenters were blind to this allocation.

**Table 1.** List of animals used for each experimental method. Number of animals and route of administration used at each age for (A) olanzapine monotherapy and modulation studies for both liquid scintillation counting and LC-MS (liquid chromatography coupled with mass spectrometry) or (B) [ ^3^H] olanzapine drug competition for liquid scintillation counting. Drugs used in drug competition experiments include: cimetidine (CIM), digoxin (DIG), fluvoxamine (FLX), lamotrigine (LTG), lithium (Li), paracetamol (PARA) and valproate (VPA). Note in pregnant experiments route of drug administration was either *i.p.* (intraperitoneal) or *i.v.* (intravenous). Value in brackets indicates number of litters used in E19 and P4 animals. Note the number of cerebrospinal fluid (CSF) samples (20) differs from brain samples (21) in A E19 acute *i.v.* to dam [ ^3^H] olanzapine group.

(A) Olanzapine monotherapy and transporter modulation studies						
Age	Route of administration	[ ^3^H] olanzapine			LC-MS	
		Acute	Prolonged	Prolonged digoxin	Acute	Prolonged
E19	*i.p.* to fetus	14 (2)	-	-	7	-
	*i.v.* to dam	21 (2)	10 (1)	10 (1)	22 (2)	10 (1)
P4	*i.p.*	12 (2)	4 (1)	5 (1)	10 (2)	6 (1)
Non-pregnant adult	*i.p.*	3	3	3	4	4

(B) [ ^3^H]-olanzapine drug competition for liquid scintillation counting								
Age	Route of administration	[ ^3^H] olanzapine						
		CIM	DIG	FLX	LTG	Li	PARA	VPA
E19	*i.v.* to dam	8 (1)	8 (1)	-	10 (1)	-	4 (1)	7 (1)
P4	*i.p.*	4 (2)	4 (2)	4 (2)	4 (2)	4 (2)	4 (2)	4 (2)
Non-pregnant adult	*i.p.*	3	3	-	3	-	3	3

### Drugs and markers

Drug doses were selected based on levels used in clinical practice (Australian Medicines Handbook, 2021) and adjusted for body weight of the animal, summarised in Table 2. Cimetidine, fluvoxamine, lithium, paracetamol and valproate were dissolved in sterile 0.9% sodium chloride solution (B. Braun, Catalogue number: 352 1370). Olanzapine was dissolved in ethanol. Digoxin and lamotrigine were dissolved in ethanol before dilution in sterile 0.9% sodium chloride solution.

**Table 2.** List of drugs used, their abbreviation, dose, supplier and catalogue number.

Drug	Abbreviation	Dose	Supplier	Catalogue #
[ ^3^H]-olanzapine	3H OLZ	Trace	American radiolabeled chemicals	ART 1788
[ ^14^C]-olanzapine	14C OLZ	Trace	American radiolabeled chemicals	ARC 3463
Olanzapine-d8	OLZ d8	Internal standard (10–100 ng/ml)	Cambridge Isotope Laboratories	O-035-1ML
N-Demethyl Olanzapine-d8	DMO d8	Internal standard (10 ng/ml)	Toronto Research Chemicals	D230972
Olanzapine	OLZ	0.15 mg/kg	Sigma Aldrich	PHR1825-1G
Cimetidine	CIM	11 mg/kg	Sigma Aldrich	C3422-5G
Digoxin	DIG	0.03 mg/kg	Sigma Aldrich	D6003-100MG
Fluvoxamine	FLX	1.5 mg/kg	Sigma Aldrich	F2802-10MG
Lamotrigine	LTG	6 mg/kg	Sigma Aldrich	PHR1392-1G
Lithium	Li	3.2 mg/kg	Sigma Aldrich	310468-100G
Paracetamol	PARA	15 mg/kg	Sigma Aldrich	A7085-100G
Valproate	VPA	30 mg/kg	Sigma Aldrich	P4543-100G

### Experimental procedure

In the acute treatment group, a single dose of 0.15 mg/kg olanzapine containing traces of [ ^3^H]-labelled olanzapine (10–20 μCi E19 dams, 1 μCi P4s, 2 μCi adults) was administered to rats by injection at E19, P4 or adult and blood, CSF and brain cortex samples were taken 30 min later. Postnatal and non-pregnant adult animals were administered olanzapine via *i.p.* injection. Pregnant animals were administered a final intravenous ( *i.v.*) dose as drug transfer from an *i.p.* injection to the fetus stops as soon as the peritoneal cavity is exposed ( Koehn *et al.*, 2019; Koehn *et al.*, 2020).

In prolonged experiments, doses of unlabelled drug were administered once (olanzapine) or twice (digoxin, *i.p.* injections, Kohen *et al.* 2019) daily for 4 days, with a final dose on the 5 ^th^ day containing [ ^3^H]olanzapine. Samples were collected 30 min post *i.p.* injection as described for the acute treatment group.

For drug competition experiments, a single injectate contained both olanzapine and one another commonly used medication: cimetidine, digoxin, fluvoxamine, lamotrigine, lithium, paracetamol or valproate together with the radiolabelled tracer [ ^3^H] olanzapine was administered and samples were collected 30 min post *i.p.* injection as described for the acute treatment group.

No animals died before completion of experiments and no results were discarded.

### Sample collection

Postnatal and non-pregnant adult animals were terminally anaesthetised 30 min after final drug injection using inhaled isoflurane (IsoFlo 100% w/w, Abbott Laboratories). Blood, brain and CSF samples were collected. Blood was collected directly from the right ventricle of the heart and CSF from the cisterna magna. Two brain samples were dissected out: cortical samples were obtained from the frontal/parietal lobes dorsal to the lateral ventricles as described previously ( Koehn *et al.*, 2019) and the brainstem.

As previously described ( Koehn *et al.,* 2019, 2020), pregnant animals were anaesthetised with *i.p.* urethane injection (25% w/v urethane, Sigma, 1 ml/100g body weight). Animals were then placed on a temperature-controlled heating pad in a supine position and an endotracheal catheter inserted to maintain a clear airway. A catheter was also inserted into the femoral artery for maternal arterial blood sampling time-matched to individual fetal collections. The cannula was flushed with 0.5 ml of heparinised saline (Hospira Inc, 5 units/ml) following each arterial blood sample. Fetal animals were exteriorised and samples serially collected starting 30 min post maternal injection. Viability of each fetus was assessed at the time of collection by observing the colour of the umbilical vessels. A final maternal blood sample was taken directly from the left cardiac ventricle and brain and CSF samples were taken following terminal exsanguination, as described above.

### Liquid scintillation counting

Samples were processed immediately after collection as previously described ( Toll *et al.*, 2021). Plasma was separated from whole blood by centrifugation (2,000xg, 5 min; Eppendorf 5453 Mini-Spin Plus centrifuge). CSF samples were also centrifuged (2,000xg, 5 min), then microscopically examined for traces of red blood cell contamination ( Habgood *et al.*, 1992). For every experiment, a sample of injectate was also measured to confirm uniformity of injected material and indicate the effectiveness of injections. Samples were weighed and transferred to scintillation vials. To solubilize brain samples, 0.5 ml Soluene350 (PerkinElmer) was added and incubated overnight at 36°C. Glacial acetic acid (2 drops, Sigma) was added to neutralize the alkaline Soluene350. All samples were mixed with 5 ml scintillant (Emulsifier-safe, PerkinElmer) and counted for 5 minutes each on a liquid scintillation counter (Tri-Carb 4910 TR, PerkinElmer) with luminescence correction on. Counts were expressed as radioactivity disintegrations per minute (DPM), blank samples containing the same tissues with no radioactivity were run alongside the samples to establish the background counts. The corresponding background counts were always subtracted from sample counts.

Sample activities were expressed as DPM/μl or mg of plasma, CSF and brain cortex tissue to normalise the values. The following equations were used to indicate the brain cortex, brainstem, CSF transfer ( Equation 1) or placental transfer ( Equation 2).

Equation 1:

$$\text{Brain or}\;\mathrm{CSF}\;\text{transfer}=\frac{\text{Cortex},\text{brainstem or}\;\mathrm{CSF}\;\mathrm{DPM}/\mu l\;\text{or}\;\mathrm{mg}}{\text{plasma}\;\mathrm{DPM}/\mu l}\times 100\%$$

Equation 2:

$$\text{Placental transfer}=\frac{\text{Fetal plasma}\;\mathrm{at}\;\text{time}\;x\;\left(\mathrm{DPM}/\mu l\right)}{\text{Average maternal plasma}\;\mathrm{up}\;\text{to time}\;x\;\left(\mathrm{DPM}/\mu l\right)}\times 100\%$$

x=fetal sampling time

### Liquid chromatography coupled with mass spectrometry (LC-MS)

The method was similar to that which has been described previously for valproate ( Toll *et al.*, 2021). Its application for measuring olanzapine is detailed here. Olanzapine-d8 (Cambridge Isotope Laboratories) and N-demethyl olanzapine-d8 (Toronto Research Chemicals) were dissolved in methanol at 100 μg/ml and 1 mg/ml as stock respectively. All stock solutions were stored at -20 °C. The internal standard mixture containing 100 pg/μl of each standard in methanol was prepared from diluting stock solutions using methanol immediately before use. To each 10 μl plasma sample, 10 μl of internal standard mixture and 80 μl of methanol were added. After mixing for 30 seconds, the sample was centrifuged at 14,000× *g* for 10 min and the top 50 μl of supernatant was transferred to a glass vial for liquid chromatography coupled with mass spectrometry (LC–MS) analysis using a Vanquish ultrahigh performance liquid chromatography (UHPLC) coupled with an Orbitrap Fusion Lumos mass spectrometer (Thermo Fisher Scientific, San Jose, CA, USA) operated at positive ion mode. Solvent A was 10 mM ammonium formate with 0.1% formic acid in water and solvent B was acetonitrile. Then, 5 μl of each sample was injected into an RRHD Eclipse Plus C18 column (2.1×1000 mm, 1.8 μm, Agilent Technology, Santa Clara, CA, USA) at 50°C at a flow rate of 350 μl/min for 1 min using 1% solvent B. During separation, the percentage of solvent B was increased from 1% to 40% in 4 min, 40% to 80% in 0.5 min, maintained at 80% for 2 min before dropping back to 1% in 0.1 min and staying at 1% for 2.4 min.

All MS experiments were performed using a heated electrospray ionization (HESI) source. The spray voltage was 3.5 kV in positive ionisation mode. The flow rates of sheath, auxiliary and sweep gases were 20 and 6 and 1 arbitrary unit(s), respectively. The ion transfer tube and vaporizer temperatures were maintained at 350°C and 400°C, respectively, and the S-Lens RF level was set at 50%. Targeted higher-energy collisional dissociation (HCD)-tandem mass spectrometry (MS/MS) scans of unlabelled and labelled olanzapine at normalized collision energy (NCE) of 35% as well as unlabelled and labelled N-demethyl olanzapine at NCE of 42% were acquired with isolation width of 4 Da, Orbitrap resolution at 15,000 (at m/z 200), maximum injection time of 22 milliseconds and automatic gain control (AGC) target of 2.5E5.

Peak areas in extracted ion chromatogram of monitored product ions from olanzapine (m/z 313.1487 to 256.0904) at 3.8 min, olanzapine-d8 (m/z 321.1989 to 260.1156) at 3.8 min, N-demethyl olanzapine (m/z 299.1330 to 198.0245) at 4.2 min and N-demethyl olanzapine-d8 (m/z 307.1833 to 198.0245) at 4.1 min were extracted using Skyline 21.2 ( RRID:SCR_014080) for quantitative analysis of drugs in each sample. Linear response range in untreated control rat plasma was tested for both unlabelled and d8-labelled olanzapine at 0.1, 1, 10, 50, 100, 200, 400 and 800 ng/ml. The ranges of linear responses were 1–800ng/ml for olanzapine and 50–800ng/ml for olanzapine-d8. ( **Extended data Figure 1**). The main olanzapine metabolite, N-demethyl olanzapine, was not detected in any of the samples tested.

### Statistical analysis

Data from all experiments are expressed as mean±standard deviation (SD). Statistical differences between cortex/plasma, CSF/plasma and fetal/average maternal plasma ratios and olanzapine measurements in plasma, CSF and brain cortex were determined using one-way ANOVA with Tukey’s posthoc test for multiple comparisons ( GraphPad Prism 9) (an open-access alternative that can perform an equivalent function is R). P≤0.05 was considered statistically significant.

## Results

### Concentration of olanzapine in rat plasma and CSF.

**Figure 1.**
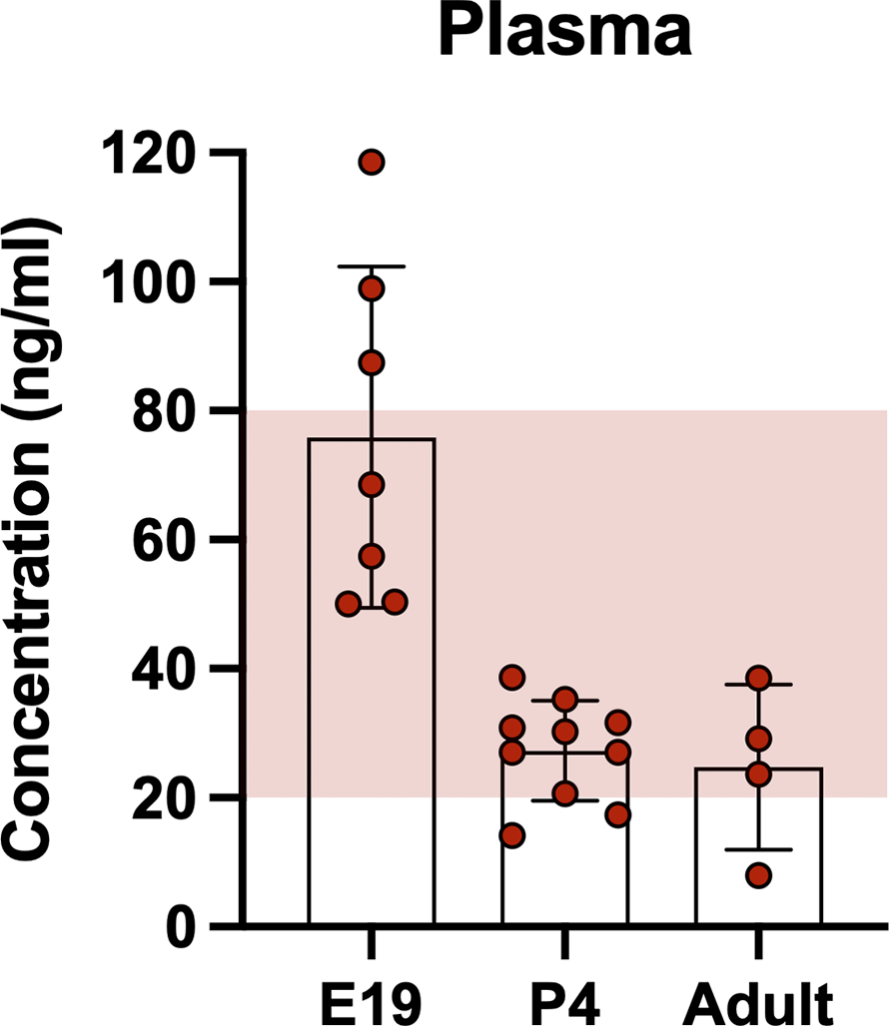
Concentration of olanzapine in rat plasma at three ages. At E19, P4 and adult 30 min after a single intraperitoneal injection of 0.15 mg/kg olanzapine its concentration was measured using liquid chromatography coupled with mass spectrometry (LC-MS). Shaded area indicates clinical therapeutic range of olanzapine (20–80ng/ml; Hiemke *et al.* 2011). Bars are means±SD. Note each point is an individual animal (n=4–10).

LC-MS was used to confirm that the injection protocol was achieving expected clinical concentrations of olanzapine in the rats’ circulating blood ( Huang *et al.*, 2022). Results are shown in Figure 1. Concentrations of olanzapine in the plasma ranged between 10 ng/ml to over 100 ng/ml with the majority of samples falling between 20–40 ng/ml. These values are within clinically accepted limits (shaded area), which in humans are 20–80 ng/ml ( Hiemke *et al.*, 2011).

### Time-course of olanzapine entry into the brain and CSF

**Figure 2.**
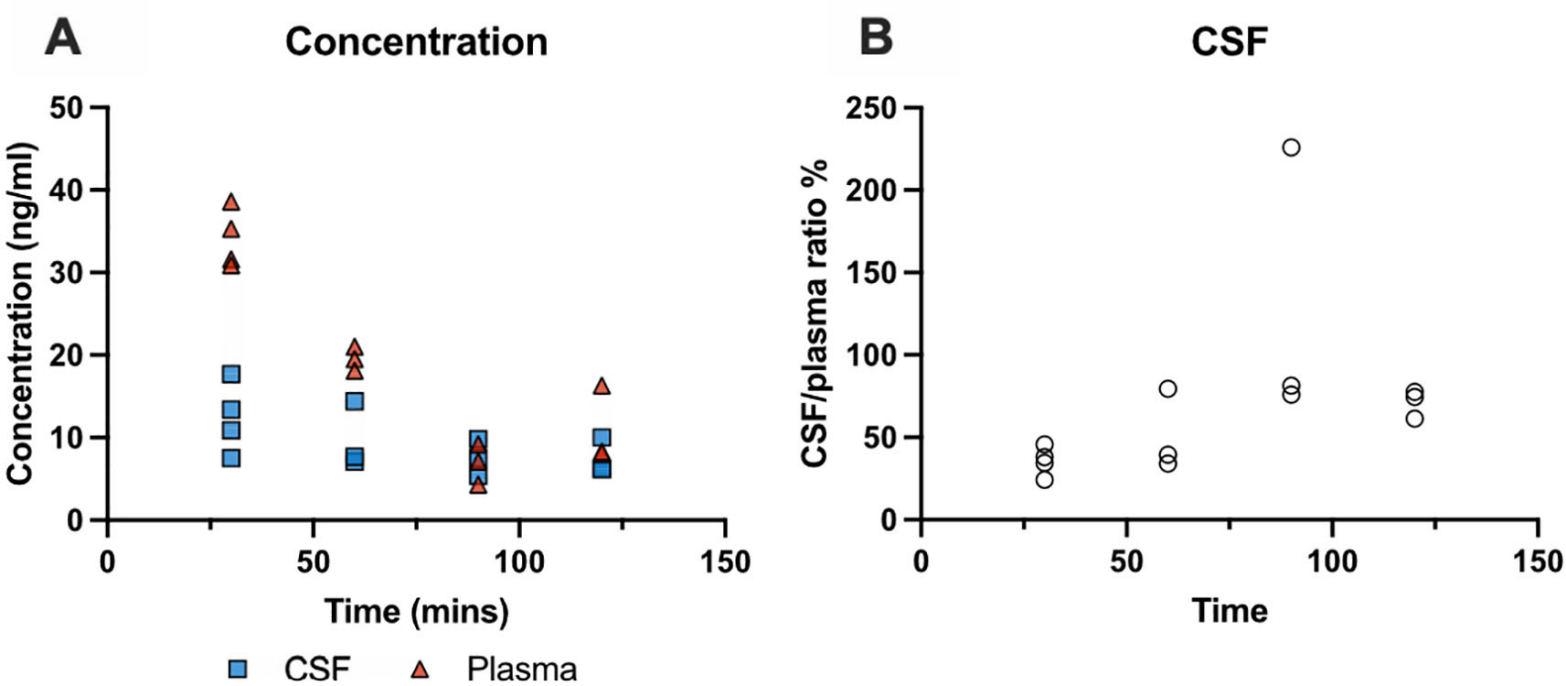
Time course of olanzapine entry into cerebrospinal fluid (CSF) in P4 rats. (A) Olanzapine concentration (ng/ml) in CSF and plasma 30 –120 min after a single intraperitoneal ( *i.p.*) injection of olanzapine (0.15 mg/kg) measured using LC-MS (liquid chromatography coupled with mass spectrometry). (B) CSF/plasma olanzapine concentration ratios (%). Note each point represents results from an individual animal (n=3–4 at each time point).

To determine the time course of olanzapine entry into the brain and CSF, P4 pups from a single litter of 13 animals were injected individually *i.p.* with a single dose of olanzapine (0.15 mg/kg) and brain, CSF and blood samples were collected at 30 min, 60 min, 90 min and 120 min. The concentration of the drug was measured by LC-MS as described in the Methods. Results are illustrated in Figure 2 and detailed in **Extended data Table 2**. Olanzapine concentrations in plasma and CSF decreased over time ( Figure 2A). In plasma, concentrations fell significantly from 34±4 ng/ml at 30 min to 11±5 ng/ml at 120 min post injection (p<0.01). CSF concentrations did not change significantly with increased exposure, remaining stable at around 9 ng/ml. CSF/plasma ratios ( Figure 2B) increased initially from around 35% to 50% then remained relatively stable between 60 and 120 min. The highest olanzapine concentration in plasma was detected at 30 min, therefore all subsequent acute experiments were performed 30 min following drug injection as in previous similar experiments ( Koehn *et al.*, 2019; Toll *et al.*, 2021).

### Dose-dependent brain and CSF entry of olanzapine

To establish if the entry of olanzapine into the brain and CSF changed depending on its dose, dose-response experiments were conducted in P4 animals. The entry of [ ^14^C]-olanzapine ( Table 2) was tested at 5 doses: 0.015 mg/kg, 0.03 mg/kg, 0.075 mg/kg, 0.15 mg/kg and 0.375 mg/kg 30 min after a single *i.p.* injection ( **Extended data Figure 2**) **.** There were no significant differences in brain and CSF entry between the doses investigated; 0.15 mg/kg was chosen for subsequent experiments as this was shown to achieve clinically relevant plasma concentrations ( Figure 1).

### Age-dependent brain and CSF entry of olanzapine

Entry of olanzapine into the CSF and brain was estimated at three ages: E19, P4 and adult using liquid scintillation counting and two injection protocols: a single dose (acute group) and multiple doses over 5 days (prolonged group) as described in the Methods. Concentration ratios (%) between the brain (cortex and brainstem) and plasma, and CSF and plasma (expressed as DPM/μl or DPM/mg of sample) were used as an index of drug’s entry ( Davson & Segal, 1996).

### Brain and CSF entry of olanzapine in acute treatment groups

**Figure 3.**
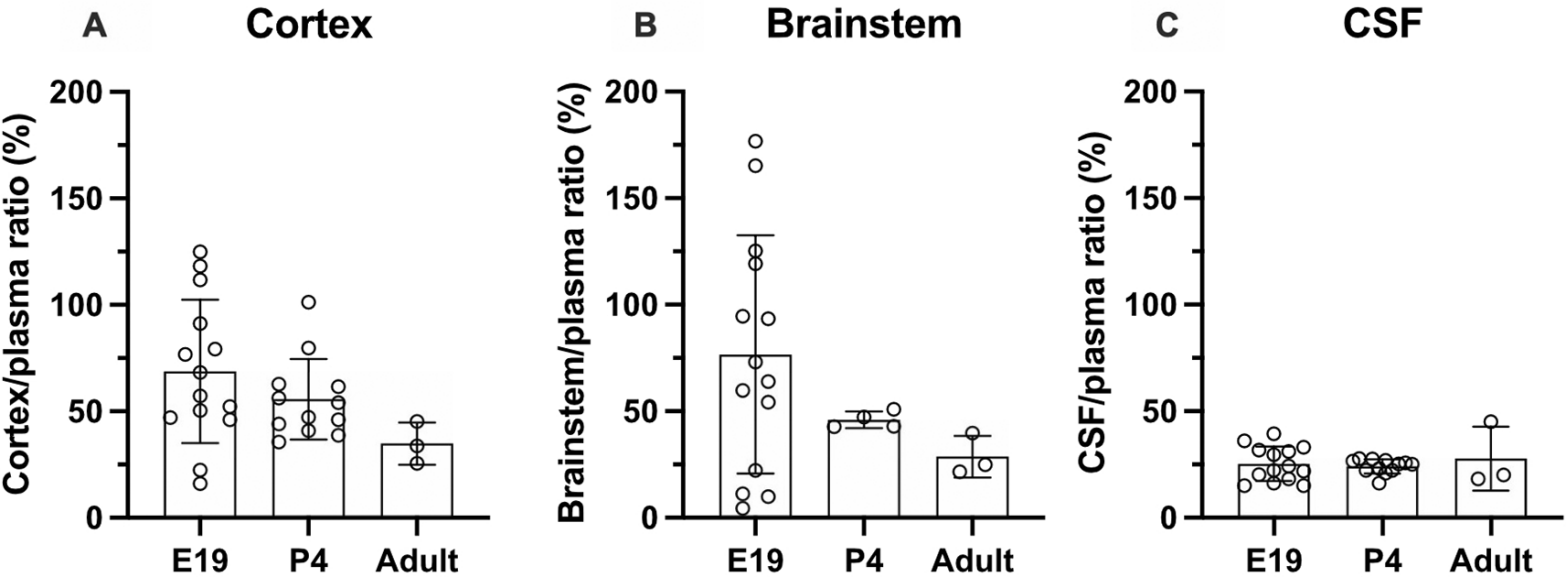
Age-dependent entry of olanzapine into the rat. (A) Brain cortex (B) brainstem and (C) cerebrospinal fluid (CSF) at three ages: E19, P4 and adult, 30 min after a single intraperitoneal ( *i.p.*) injection of 0.15 mg/kg olanzapine containing a radioactive tracer ([ ^3^H]olanzapine). Bars are means±SD. Note each point represents results from an individual animal (n=3–14).

Results of experiments measuring the entry of [ ^3^H]-olanzapine into the brain and CSF in acute experiments are illustrated in Figure 3 and numerical values are shown in **Extended data Table 2.** Brain entry of the drug into both the cortex and brainstem was highly variable, especially in fetal brains, and therefore no significant difference between the ages was detected. The values ranged from under 10% to over 100% at E19 and around 50% in postnatal animals ( Figure 3). In contrast, values obtained for the CSF’s entry were more stable at around 25% and were similar at all three ages.

### Brain and CSF entry of olanzapine following prolonged treatment

**Figure 4.**
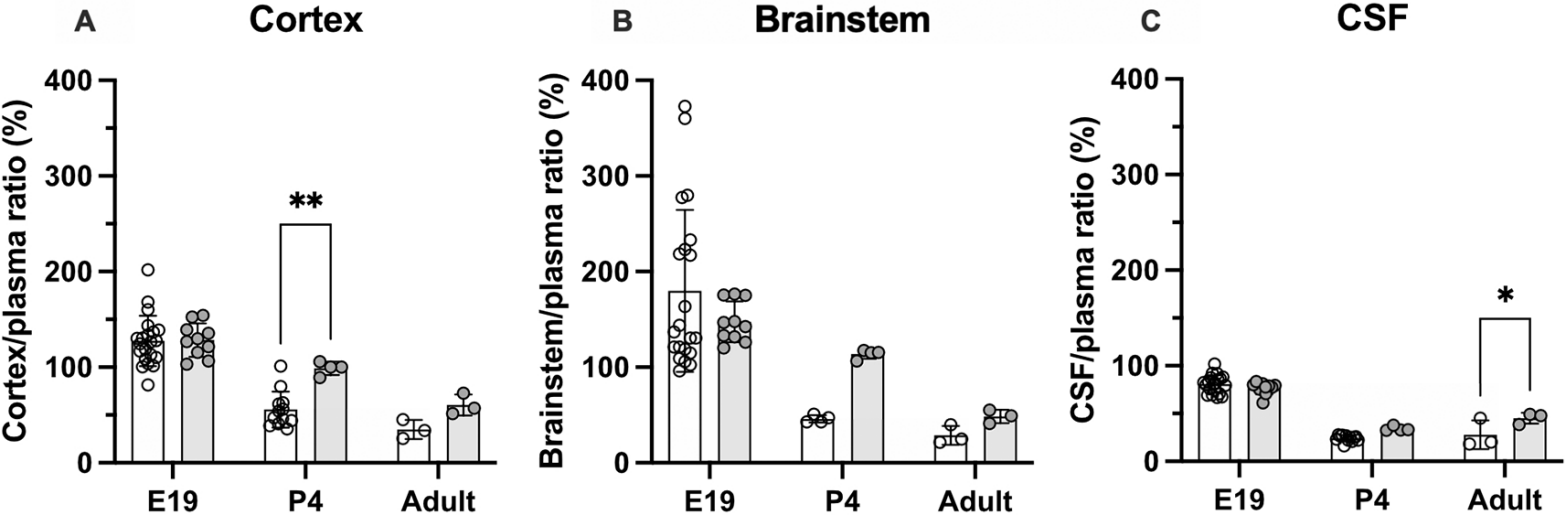
Comparison of acute (white bars) and prolonged (grey bars) olanzapine treatment. (A) Brain cortex/plasma, (B) brainstem/plasma and (C) cerebrospinal fluid (CSF)/plasma olanzapine concentration ratios (%) in the rat at three ages: E19, P4 and adult. Final injection included a radioactive tracer ([ ^3^H]olanzapine). Final intravenous ( *i.v.*) dose was given maternally for E19 animals. Bars are means±SD. Note each point is an individual animal (n=3–10). *p<0.05, **p<0.01.

Entry of olanzapine was also investigated in animals that received doses of the drug over several days. Olanzapine (0.15 mg/kg) was given once daily *i.p.* for 5 days and on the final day trace amounts of [ ^3^H]-olanzapine) were added to the dose (see Methods). For the fetal animals, dams were given the olanzapine treatments *i.p.* starting at E15, then on the final day at E19, the last dose was given *i.v..* Results are displayed in Figure 4 and numerical values shown in **Extended data Table 3**. In postnatal animals, prolonged treatment resulted in some significant changes in olanzapine entry into both the brain and CSF. At P4 olanzapine entry into the cortex increased from 56±19% in the acute group to 99±7% in the prolonged group (p<0.01), while transfer into the adult CSF increased from 28±15% in the acute group to 45±6% in the prolonged group (p<0.0.5). There were no significant changes in the E19 pups following prolonged olanzapine exposure (p>0.05).

### Placental transfer of olanzapine in acute and prolonged experiments

**Figure 5.**
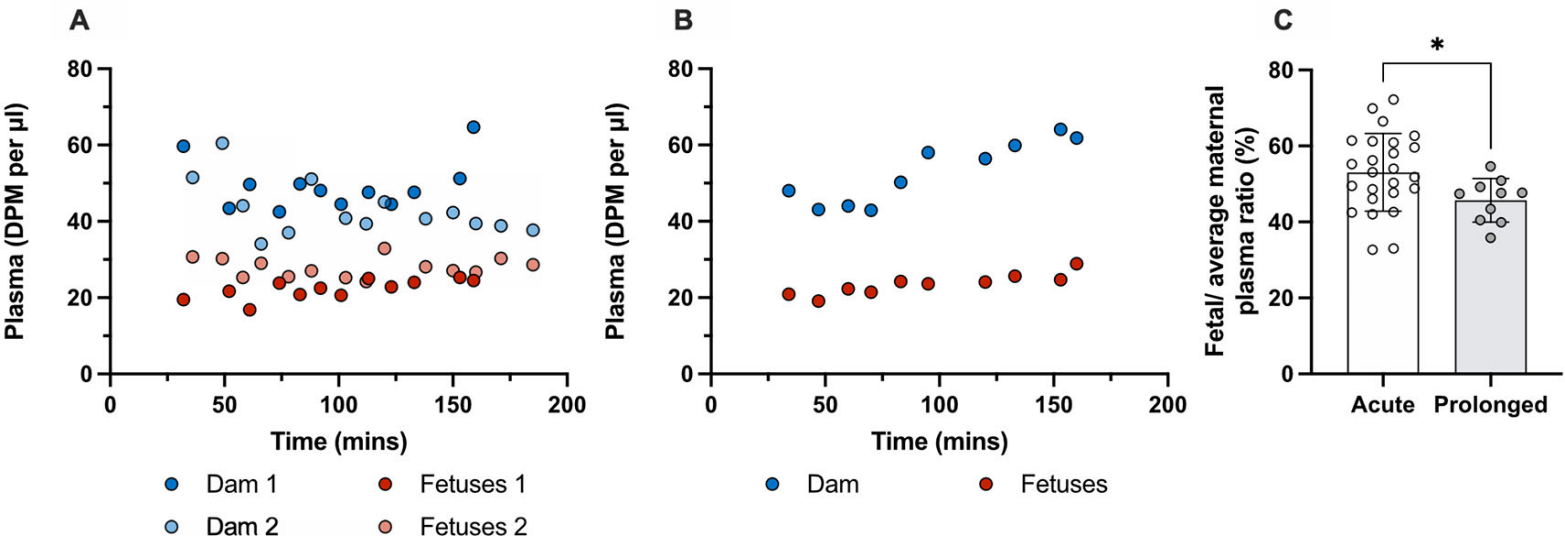
Counts (DPM/μl) in plasma of dams and their fetuses at E19. (A) A single intravenous ( *i.v.*) injection of 0.15 mg/kg olanzapine with a radioactive tracer ([ ^3^H]olanzapine) to each dam and (B) counts (DPM/μl) in plasma after 4 daily intraperitoneal ( *i.p.*) injections of 0.15mg/kg olanzapine followed by olanzapine with a radioactive tracer ([ ^3^H]olanzapine) *i.v.* on the 5 ^th^ day. (C) Placental transfer as calculated by fetal/average maternal plasma ratio (%) using results from A and B. Bars are means±SD. Note dam values are serial plasma samples taken between 30–160 min post maternal injection from one or two individual dams, fetal values and ratios are from individual animals (n=10–25). *p<0.05.

Placental transfer of olanzapine between the dam and the fetus at E19 was estimated by comparing levels of the drug in fetal and maternal plasma ( Equation 2) in both the acute and prolonged ( **Extended data Table 3**) experiments. Results are displayed in Figure 5.

In acute experiments the transfer of olanzapine across the placenta from maternal into the fetal circulation was 53±10% (n=25).

Placental transfer of olanzapine following prolonged treatment was 49±4% (n=10). Thus, prolonged olanzapine treatment significantly decreased placental drug transfer compared to the acute treatment group (p<0.05) but only to a small extent. Results are presented in Figure 5 and **Extended data Table 3**.

### Pharmaceutical modulation of olanzapine transfer

Two approaches were used to investigate if transfer of olanzapine across brain and placental barriers can be influenced by (i) modulating the function of an ABC transporter, P-glycoprotein (Pgp) that has been suggested to be involved in olanzapine transfer ( Boulton *et al.*, 2002; Wang *et al.*, 2004) or (ii) by co-treatment with other drugs that could compete with olanzapine for the same transporter, including digoxin, a substrate for Pgp ( Mayer *et al.*, 1997) and cimetidine, a substrate for breast cancer resistant protein (BCRP, Pavek *et al.*, 2005; Staud *et al.*, 2006).

### Transporter modulation by digoxin in acute and prolonged experiments

**Figure 6.**
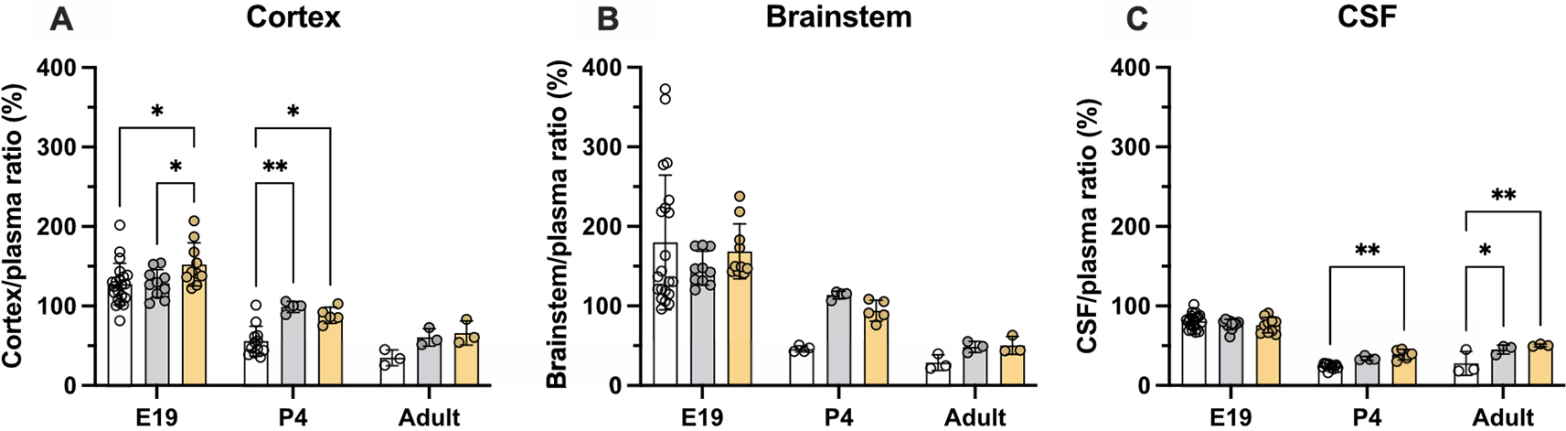
Comparison of olanzapine concentration ratios (%) in the rat. (A) Brain cortex/plasma, (B) brainstem/plasma and (C) cerebrospinal fluid (CSF)/plasma at E19, P4 and adult after a single intravenous ( *i.v.*) injection of 0.15 mg/kg olanzapine (acute, white bar), with five day daily intraperitoneal ( *i.p.*) 0.15 mg/kg olanzapine treatment (prolonged, grey bars) or 0.03 mg/kg digoxin *i.p.* twice daily for 5 days (digoxin, yellow bars). In all experiments samples were collected 30–185min after the final maternal *i.v.* injection containing olanzapine with a radioactive tracer ([ ^3^H]olanzapine). Bars are means±SD. Note each point is an individual animal (n=3–10). *p<0.05, **p<0.01

Transfer of olanzapine across the placenta at E19 and its entry into the brain and CSF was investigated following either single (acute, data from Figure 4) or repeated (prolonged) administration including co-administration with digoxin, a known ABC transporter modulator. It was shown previously that this treatment regime in rats was able to upregulate *pgp* expression and reduce digoxin entry into the brain ( Koehn *et al.*, 2019). In the fetal animals, prolonged digoxin treatment significantly increased olanzapine transfer into the brain (152±27%, n=10) compared to prolonged olanzapine alone (128±18%, n=10, p<0.05). At P4, compared to acute treatment, prolonged administration of either olanzapine or digoxin increased olanzapine transfer into the brain (from 56±19% to ~95%, p<0.01 and p<0.05 respectively) whereas only repeated digoxin treatment increased transfer in the CSF (from 24±3% to 39±7%, p<0.05). In the adult, prolonged treatment with either olanzapine (61±11%) or digoxin (66±15%) increased olanzapine transfer into the CSF compared to acute treatment alone (35±10%, p<0.05 and p<0.01 respectively). Results are illustrated in Figure 6 and listed in **Extended data Table 4**.

**Figure 7.**
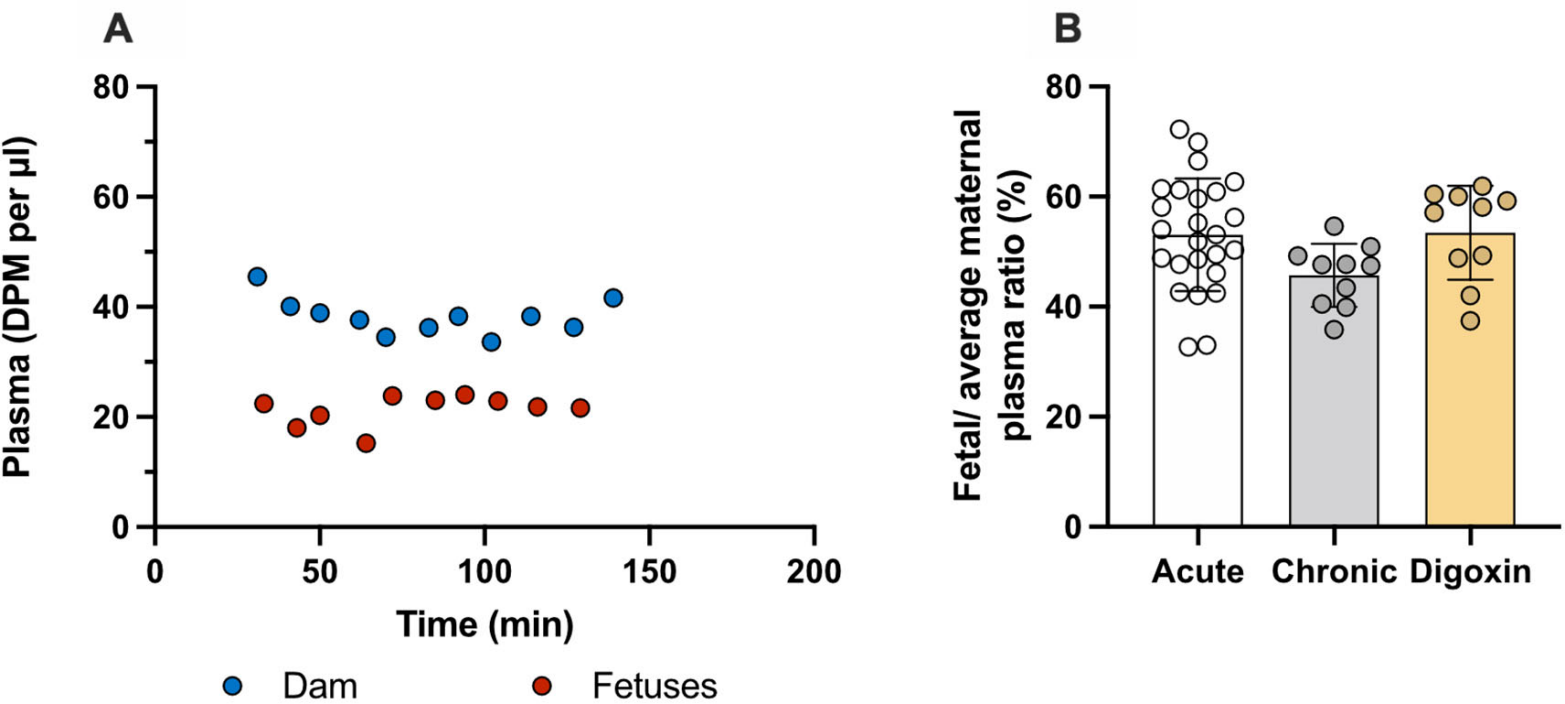
Prolonged daily treated dam and fetuses at E19. (A) Plasma counts (DPM/μl) after twice daily intraperitoneal ( *i.p.*) injections of 0.03mg/kg digoxin for 4 days and olanzapine with a radioactive tracer ([ ^3^H]olanzapine) intravenous ( *i.v.*) on the 5 ^th^ day. (B) Placental transfer displayed as fetal/average maternal plasma ratio in the E19 rat after *i.v.* injection of 0.15 mg/kg olanzapine (acute, white bar; data from Figure 6), four day daily *i.p.* 0.15 mg/kg olanzapine treatment (prolonged, grey bars; data from Figure 7) or 0.03 mg/kg digoxin *i.p.* twice-daily for 4 days (digoxin, yellow bars). Samples collected 30–185 min after the final maternal *i.v.* injection containing olanzapine with a radioactive tracer ([ ^3^H]olanzapine) for all groups. Bars are means±SD. Note each point is an individual animal (n=10).

Transfer of olanzapine across the placenta from dam to fetus was unchanged in the prolonged digoxin treated animals with placental transfer remaining at around 50%. Results are presented in Figure 7 and **Extended data Table 4**. Entry of olanzapine following either prolonged olanzapine or digoxin treatment appeared to follow a similar pattern, potentially suggesting a shared mechanism of entry.

### Drug competition

**Figure 8.**
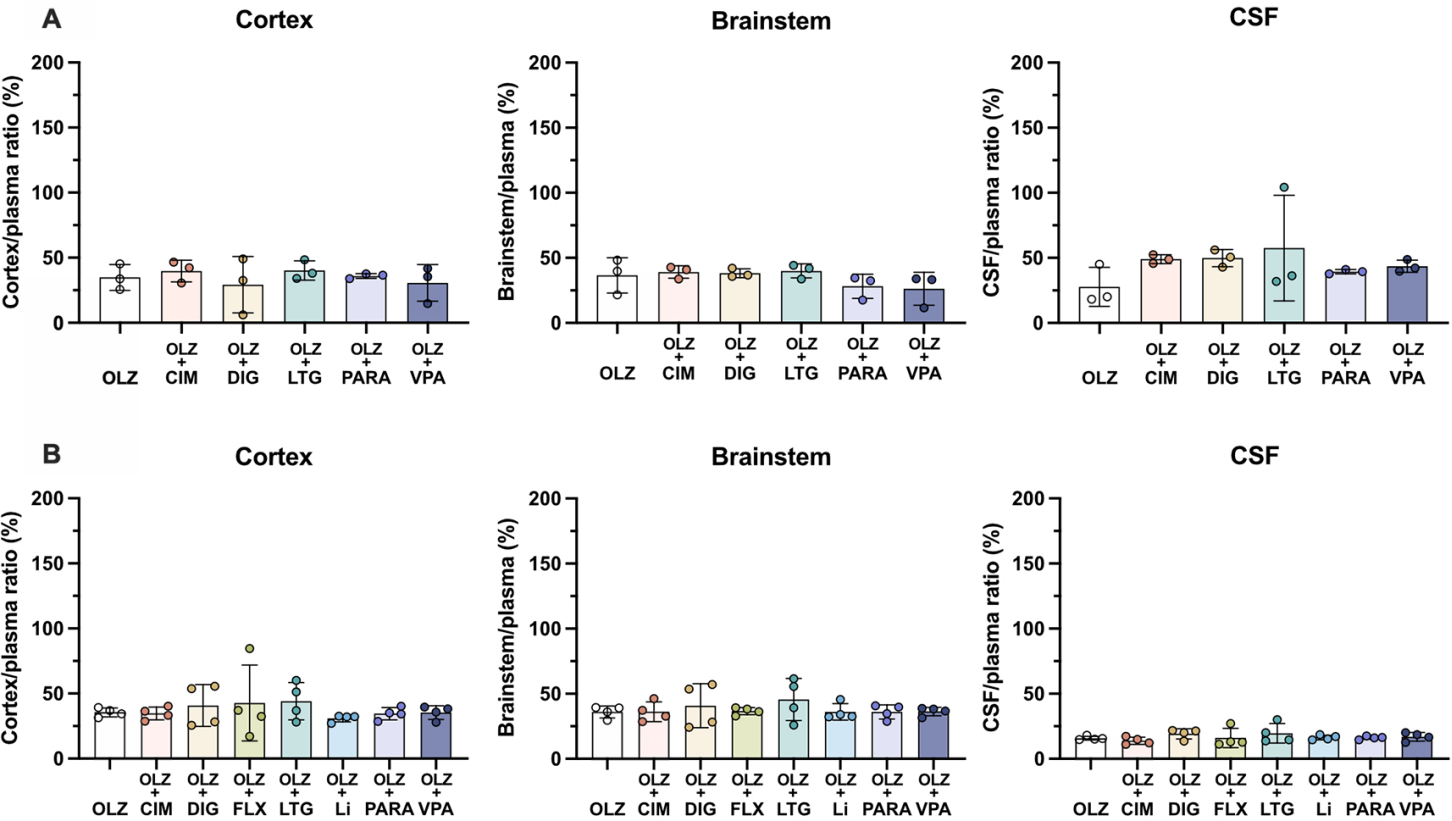
Entry of [ ^3^H]-olanzapine into the (A) non-pregnant adult and (B) postnatal day 4 rat brain cortex, brainstem and cerebrospinal fluid (CSF). Single intraperitoneal ( *i.p.*) injection either as monotherapy or in combination with: cimetidine (CIM, 11 mg/kg), digoxin (DIG, 0.03 mg/kg), lamotrigine (LTG, 6 mg/kg), paracetamol (PARA, 15 mg/kg) or valproate (VPA, 30 mg/kg). Bars are means±SD. Note each point is an individual animal (n=3–4).

**Figure 9.**
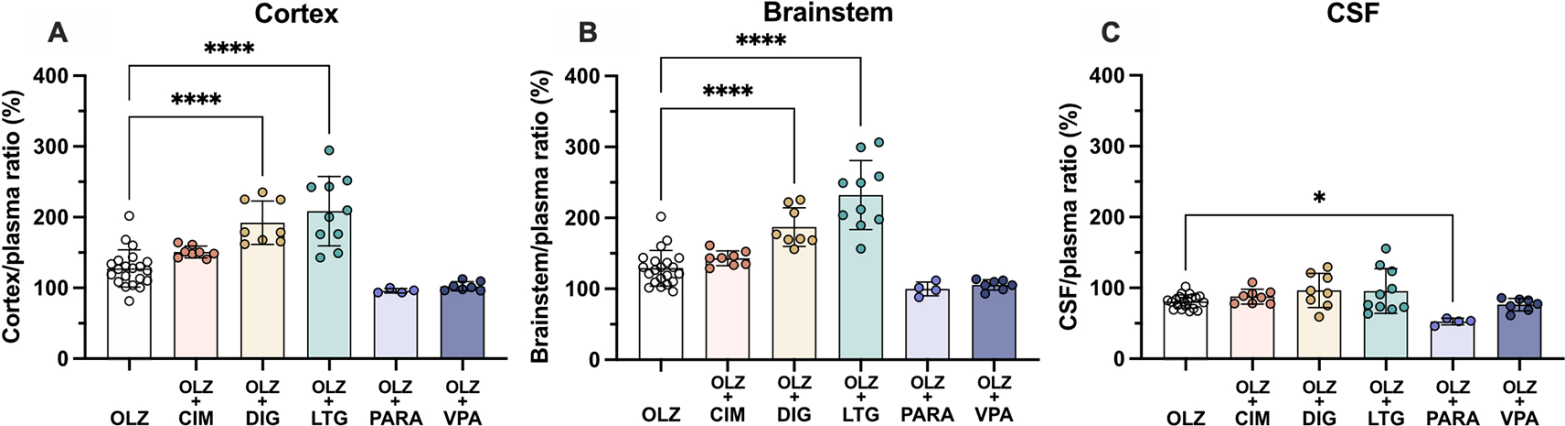
Entry of [ ^3^H]-olanzapine into the E19 rat. (A) Brain cortex, (B) brainstem and (C) cerebrospinal fluid (CSF) after a single intravenous ( *i.v.*) injection either as monotherapy or in combination with: cimetidine (11 mg/kg), digoxin (DIG, 0.03 mg/kg), lamotrigine (LTG, 6 mg/kg), lithium (Li, 3.2 mg/kg), paracetamol (PARA, 15 mg/kg) or valproate (VPA, 30 mg/kg). Bars are means±SD. Note each point is an individual animal (n=4–21). *p<0.05, ****p<0.0001.

**Figure 10.**
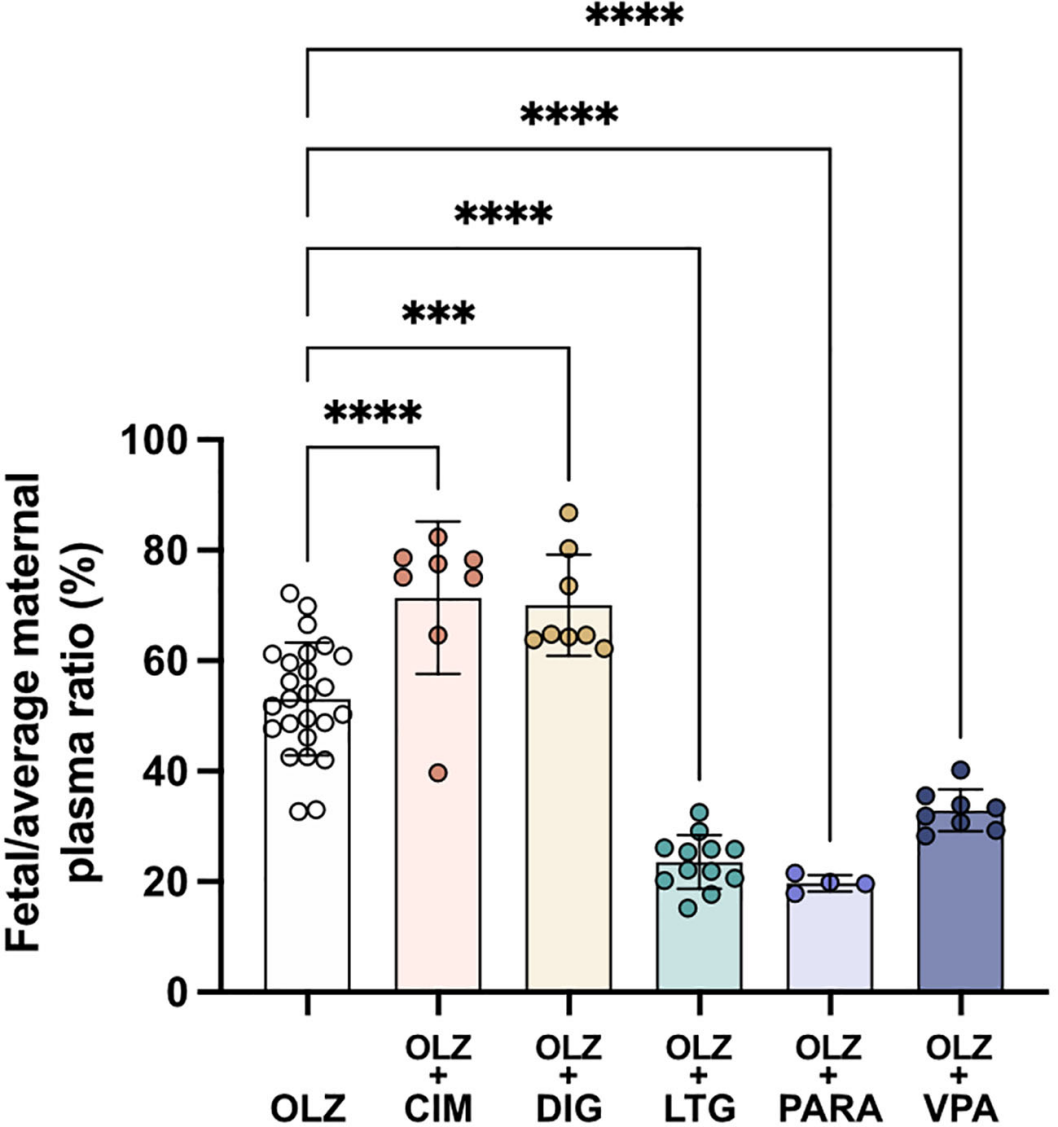
Transfer of [ ^3^H]-olanzapine across the placenta. Calculated by fetal/average maternal plasma ratio (%) after a single intravenous ( *i.v.*) injection either as monotherapy or in combination with: cimetidine (CIM, 11 mg/kg), digoxin (DIG, 0.03 mg/kg), lamotrigine (LTG, 6 mg/kg), paracetamol (PARA, 15 mg/kg) or valproate (VPA, 30 mg/kg). Bars are means±SD. Note each point is an individual animal (n=4–26). ***p<0.001, ****p<0.0001.

To investigate the effects of co-administration of several common medications on olanzapine transfer, seven drugs (cimetidine, digoxin, fluvoxamine, lamotrigine, lithium, paracetamol and valproate, Table 2) were given acutely each in combination with olanzapine. Transfer of olanzapine into the rat brain, CSF and across the placenta was measured at E19, P4 and adult. All drug competition results are shown in Figures 8– 10 and in **Extended data Table 5**.

In postnatal animals, there was no significant difference in olanzapine transfer after combination therapy with any of the drugs investigated. In the non-pregnant adults, olanzapine transfer remained at around 30–50% in both brain and CSF ( Figure 8). At P4 there was also no significant difference in olanzapine transfer after combination therapy and its entry into the brain and CSF remained stable at around 30–40% and 10–20% respectively ( Figure 8).

However, in fetal animals at E19 co-treatment with either digoxin (192±31%, n=8) or lamotrigine (209±49%, n=10) increased olanzapine transfer into the fetal brain cortex compared to olanzapine alone (128±26%, n=21, both p<0.0001). This trend was also seen in the brainstem, where digoxin and lamotrigine combination therapy also increased olanzapine permeability compared to olanzapine monotherapy (from 129±25%, n=21, to 187±27%, n=8, or 232±49%, n=10 respectively, both p<0.0001). In the CSF, combination therapy with paracetamol decreased olanzapine transfer from 81±9% (n=20) in olanzapine monotherapy to 53±5% (n=4) in combination therapy, p<0.05 ( Figure 9). The other drugs co-administered with olanzapine did not appear to affect its entry at this age.

Transfer of olanzapine across the placenta from maternal to fetal circulation changed significantly following co-administration with all of the drugs investigated ( Figure 10). Cimetidine and digoxin co-treatment increased olanzapine transfer across the placenta from 53±10% to around 70% (both n=8, p<0.0001 and p<0.001 respectively) whereas lamotrigine, paracetamol or valproate all decreased olanzapine placental transfer to 24±5%, 20±2% and 33±4% respectively (n=4-12, all p<0.0001; Figure 10). Thus, it appears that both influx and efflux transporters are involved in olanzapine transfer across the placental barrier and these can be influenced by different therapeutics (see Discussion).

## Discussion

In this study, we aimed to determine the extent of olanzapine transfer across the placenta during late pregnancy and its entry into the brain at different stages of development in monotherapy as well as in combination with several medications that are often prescribed with olanzapine. Olanzapine transfer was determined by measuring radiolabelled tracers in plasma, CSF, brain cortex and brainstem in the rat at three developmental ages: late-gestation fetus (E19), early postnatal pup (P4) and adult following either single or repeated doses of the drug. Levels of the drug in blood plasma were confirmed by LC-MS.

Drug transfer into the brain and CSF *in vivo* is determined by multiple factors including protein binding, lipid solubility and cellular transport mechanisms. Olanzapine has been reported to be ~93% protein bound in humans with binding being concentration independent ( Kassahun *et al.*, 1997). However, in our preliminary rat studies we found that olanzapine appears to be less protein bound (~40%) at all ages tested from E19 to adults, but was also concentration independent (F.Qiu, personal communication). As the unbound drug is able to pass across brain and placental barriers more readily than their protein-bound counterparts ( Robinson & Rapoport, 1986; Griffiths & Campbell, 2015; Polin *et al.*, 2017) the increase in available free drug potentially indicates that more olanzapine is available for transport/transfer across barriers in rats. Olanzapine is highly lipid soluble with a predicted LogD of 1.9–2.8 at pH 7.4 ( Tetko *et al.*, 2005; ChemSpider, 2022). However, it appears that olanzapine permeability does not solely depend on its protein binding capacity or lipophilicity as its brain entry is higher compared to another antipsychotic drug, risperidone, which has lower protein binding and a similar logD ( Mannens *et al.*, 1994; Dave and Morris, 2016).

The major transporters involved in mediating drug movement across placental and brain barriers include adenosine triphosphate binding cassette (ABC) efflux transporters as well as selective bi-directional solute carriers (mostly influx transporters, SLCs). ABC efflux transporters of note include P-glycoprotein (Pgp), breast cancer resistance protein (BCRP) and multidrug resistance proteins (MRPs); the SLCs facilitating drug transfer include the SLC22A and SLCO superfamilies ( Ghersi-Egea *et al.*, 2018). Olanzapine has been classified as an intermediate P-glycoprotein (Pgp) substrate with high specificity, but low maximal capacity ( Boulton *et al.*, 2002). The entry of olanzapine into brain was tested by Wang *et al.* (2004) using Pgp knockout mice. In that study, mice were injected *i.p.* with 2.5 mg/kg of olanzapine, with samples collected one hour after injection. Whilst the plasma concentration remained relatively constant, brain concentrations increased in the knockout group compared to wildtype, implicating a link between Pgp and olanzapine.

Effectiveness of these transporters can also be modulated by inducers and inhibitors. In the case of efflux transporters inducers increase the capacity of the transporter thereby reducing drug entry whereas inhibitors decrease transporter- mediated efflux therefore increasing drug entry. The inverse is true for influx transporters. One such example of transporter inhibition includes competition for the same transporter via drug-drug interactions when more than one substrate drug is present ( Ekford and Sharom, 2006; Wessler *et al.*, 2013). In the present study, olanzapine entry into brain was also assessed when challenged with other therapeutics in both single or repeated administration to confirm its relationship with Pgp and investigate any potential interactions with other transporters (see below).

### Olanzapine transfer across brain barriers in monotherapy

In monotherapy, entry of olanzapine into the brain or CSF was similar in all three age groups when the drug was administered acutely ( *i.p.*) to individual rats ( Figure 3). There was also no difference in its entry into different parts of the brain (cortex and brainstem). Prolonged treatment increased olanzapine entry into both brain and CSF in postnatal animals (P4 and adult), but not at E19 ( Figure 4). However, at E19, brain entry was already at 100% of plasma levels in the cortex, brainstem and CSF, indicating entry was not limited by ABC transporter efflux (Kohen *et al.*, 2019).

The levels of olanzapine measured were variable between individual animals making statistical age-comparisons difficult. Such variability has previously been reported by Aravagiri *et al.*, 1999, who showed that olanzapine passes through the blood brain barrier and accumulates in the brain. Rats chronically exposed to 0.25 to 6 mg/kg/day oral or *i.p.* doses of olanzapine were reported to have brain/plasma concentration ratios which ranged from 540% to 1,760% following *i.p.* injections and 630% to 1,310% following oral administration ( Aravagiri *et al.*, 1999). In contrast, in a human study involving patients who had taken 2.5–25 mg/day oral doses of olanzapine for 0.2 to 11 years, entry into the CSF was only 12% of serum levels ( Skogh *et al.*, 2011). This pattern of higher brain entry compared to CSF was also observed in this study, especially in the younger animals ( Figure 4).

### Effects of upregulation of Pgp expression on olanzapine transfer across brain barriers

Digoxin has previously been used to determine if brain entry of olanzapine is affected by co-administration with a known Pgp substrate ( Mayer *et al.*, 1997). In a more recent study, repeated digoxin treatment increased *abcb1a* (Pgp) expression in the adult cortex by 1.21-fold and was accompanied by a 7% decrease in digoxin entry into the adult brain compared to a single dose treatment group ( Koehn *et al.*, 2019). In the present study, the same prolonged dosing protocol was utilised to induce Pgp expression and [ ^3^H]-olanzapine was included with the final dose to measure its transfer. An increase in olanzapine transfer into the P4 brain and adult CSF was observed following both prolonged olanzapine and prolonged digoxin treatments, potentially indicating that these drugs share the same mechanism of limiting entry; this supports previous studies suggesting their role as Pgp substrates ( Mayer *et al.*, 1997; Boulton *et al.*, 2002; Wang *et al.*, 2004). However, in this case, the effect was an increase in olanzapine transfer, which may be a consequence of the dose of drugs used being too high and thus exceeding the efflux capacity of the transporter at the brain barriers in spite of its increased expression. A similar observation was described previously for paracetamol ( Koehn *et al.*, 2019)

### Effects of combination therapy on olanzapine entry across brain barriers

Olanzapine is routinely prescribed as a monotherapy to treat psychiatric disorders, but in many patients, additional medications are also used. Drugs are commonly taken in combination with other therapeutics to either increase their efficacy of treatment (e.g. Tohen *et al.*, 2002) or to treat co-existing conditions. To investigate the potential effects of other therapeutics on olanzapine brain entry, a combinatory approach was taken. When considering the effects of drug combination therapy on permeability, increased entry is generally interpreted to reflect competition for the same efflux transporter ( Ekford & Sharom, 2006; Wessler *et al.*, 2013), whereas a decrease is thought to reflect competition for the same influx mechanism ( Kell, 2021). Olanzapine transfer into the brain and CSF at three developmental ages was measured following co-administration with one of seven medications (see Methods) selected for their peripheral (cimetidine and digoxin) or central nervous system (fluvoxamine, lamotrigine, lithium, paracetamol and valproate) mode of action.

Transport of drugs is multifaceted with their transfer limited by multiple transporters with effectiveness being both barrier- and age-dependent ( Ek *et al.*, 2010). Two peripherally acting drugs, cimetidine and digoxin are known to be BCRP and Pgp substrates respectively ( Mayer *et al.*, 1997). The antidepressant fluvoxamine, a serotonin reuptake inhibitor (SSRI), also appears to be a Pgp substrate although with some discrepancy between studies ( O’Brien *et al.*, 2012). Currently, transport mechanisms for the analgesic paracetamol are yet to be determined. However, its metabolites: glucuronide-, sulfide- and glutathione-conjugated paracetamol have been found to be transported by MRP1, 2 and 4 in the liver ( Koenderink *et al.*, 2020). Lamotrigine and valproate are both used to treat epilepsy and bipolar disorder, but the mechanisms limiting their entry into brain differ. Lamotrigine, has been indicated as a substrate for efflux transporters Pgp ( Potschka *et al.*, 2002) and BCRP ( Römermann *et al.*, 2015) as well as the influx transporter *slc22a1* (OCT1; Dickens *et al.*, 2012). Valproate efflux appears to be mediated by Pgp and MRP4 in the placenta ( Jinno *et al.*, 2020), but not in the brain ( Baltes *et al.*, 2007). Valproate shows limited entry into brain, especially in the adult ( Toll *et al.*, 2021), which is likely due to its polar nature (LogD at pH 7.4 is 0.1-0.5, Chemspider, Tetko *et al.*, 2005). However, there is evidence of some inward transport mediated via slc16a1 (MCT1; Vlieghe and Khrestchatisky, 2013).

Lithium, commonly used to treat bipolar disorder, was also included in the drug competition group as a biological internal control. Being an ion, lithium enters the brain by a combination of passive diffusion ( Wraae, 1978), transfer through ion channels and by ion transporters ( Luo *et al.*, 2018). It is therefore unlikely to compete with olanzapine for facilitated entry into or efflux from the brain. Entry of lithium into the brain and across the placenta has been previously described in Chiou *et al.*, 2021. At postnatal day 4, entry of olanzapine into the brain and CSF was similar when administered as a monotherapy or in combination with lithium confirming no effect of lithium on olanzapine transfer. Furthermore, at P4 and adult there were no significant changes in olanzapine transfer into either brain or CSF when administered in combination with any of the other drugs investigated. These findings are in agreement with studies by Jann *et al.* (2006) and Sidhu *et al.* (2006) who reported that co-administration of olanzapine and lamotrigine in healthy human adults did not influence the entry of either drug.

In contrast to the postnatal animals, at E19 co-administration of olanzapine with other medications did affect olanzapine entry. Digoxin and lamotrigine co-treatment both increased olanzapine entry into the fetal brain, indicating competition for the same efflux transporter(s). On the other hand, co-treatment with paracetamol decreased olanzapine entry into the fetal CSF, suggesting competition for the same influx transporter(s). The observed increased olanzapine entry into the fetal brain following combination with fellow Pgp substrates digoxin and lamotrigine, is consistent with our previous findings of lower expression of *abcb1a* ( *pgp)* in fetal rats compared to adult animals ( Ek *et al.*, 2010; Koehn *et al.*, 2021), implying less capacity of the fetal brain to efflux Pgp substrates.

### Placental transfer of olanzapine

Some human studies have investigated the placental transfer of olanzapine, either using *ex vivo* models or at birth using maternal and umbilical blood. Placental passage of olanzapine from the maternal to fetal compartment was reported to be 5–14% in a study using human placental explants perfused with 10 ng/ml olanzapine over a 4-hour period ( Schenker *et al.*, 1999). In contrast, an *in vivo* human study analysing the olanzapine concentration in the mother’s plasma compared to plasma from the umbilical cord of the baby at delivery reported 72% transfer. Mothers in this study took olanzapine (mean 8.9 mg/day at delivery) for a minimum of 2 weeks before delivery ( Newport *et al.*, 2007). Previous studies have reported that the rate of congenital malformation in babies whose mothers have taken olanzapine during pregnancy is no different from the background incidence ( Brunner *et al.*, 2013). However, there are still no controlled studies investigating the use of olanzapine during pregnancy in humans probably due to ethical concerns.

In this project, using an animal model, transfer of olanzapine across the placenta was measured by comparing its concentration in fetal plasma and maternal plasma in time matched sample pairs ( ***Methods***). Following a single dose, olanzapine placental transfer was around 55% and decreased to around 45% following repeated treatment, suggesting an upregulation of efflux mechanisms induced by repeated exposure to the drug. In acute combination experiments, decreased transfer of olanzapine across the placenta was observed following co-treatment with lamotrigine, paracetamol or valproate ( Figure 10). However, in a reverse experiment observing the effects of olanzapine co-treatment on transfer of other medications, paracetamol and valproate transfer across the placenta was increased following olanzapine combination therapy (unpublished work, Huang *et al.*, 2023 in preparation). These increases in fetal drug exposure with co-treatment could be of clinical concern due to recent controversies surrounding paracetamol’s safety during pregnancy ( Masarwa *et al.*, 2020; Parker and Werler, 2020), while valproate is a known teratogen ( Tomson *et al.*, 2018; Abou-Khalil, 2019; Vajda *et al.*, 2012).

### Comparison of maternal blood levels to fetal brain levels of olanzapine in mono and combination therapies

**Figure 11.**
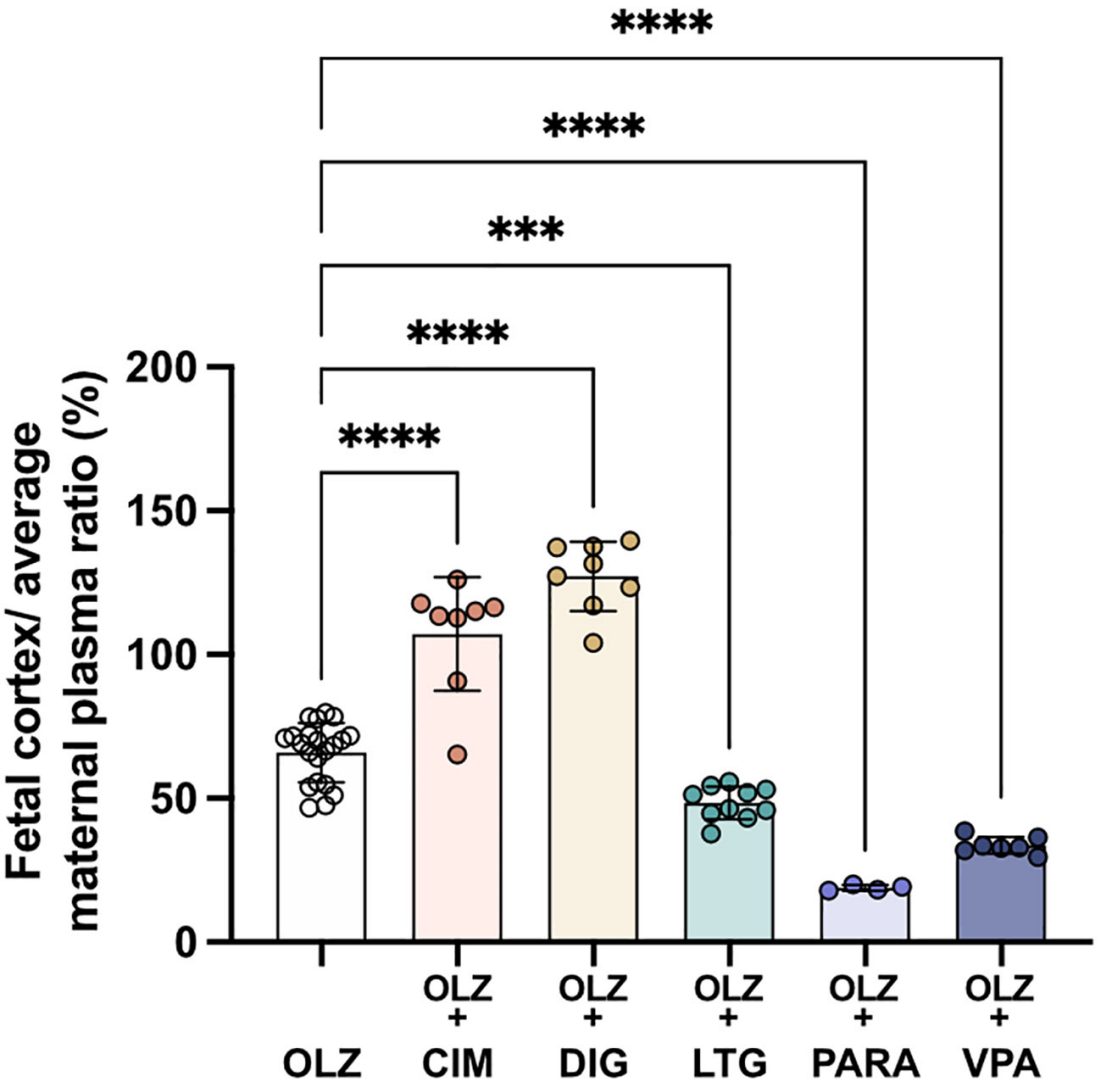
Transfer of [ ^3^H]-olanzapine across the placenta and into the fetal brain cortex. Calculated by fetal cortex/average maternal plasma ratio (%) after a single intravenous ( *i.v.*) injection either as monotherapy or in combination with: cimetidine (CIM, 11 mg/kg), digoxin (DIG, 0.03 mg/kg), lamotrigine (LTG, 6 mg/kg), paracetamol (PARA, 15 mg/kg) or valproate (VPA, 30 mg/kg). Bars are means±SD. Note each point is an individual animal (n=4–21). ***p<0.001, ****p<0.0001.

Entry of olanzapine into the fetal brain can be estimated by measuring amounts of radioactivity-traced olanzapine in fetal brain compared to its levels in maternal plasma ( Figure 11). This appears to be predominantly determined by placental transfer. For example, inclusion of lamotrigine decreased olanzapine entry from fetal blood into the cortex slightly but significantly (from 66±10%; n=21 to 58±6%, p<0.001; n=10) but there was an overall decrease due to the more substantial decrease in placental transfer (from 53±10% to 24±5%; n=10-21). Paracetamol or valproate combination therapy also decreased olanzapine transfer across the placenta to 19±1% (p<0.0001; n=4) and 34±3% (p<0.0001; n=7) respectively. In contrast to the decrease in placental olanzapine transfer that occurred in the presence of paracetamol or valproate, co-treatment with cimetidine or digoxin resulted in increased olanzapine placental transfer exceeding 100%, indicating olanzapine accumulates to a higher level in fetal brain than in maternal plasma.

### Limitations of the study

1. Drug levels contained within the blood vessels of brain tissue (i.e. residual vascular space) were not taken into account in the present study. Vascular space in the rat brain from E16 to adult has been reported to be around 2–4% ( Toll *et al.*, 2021, Qiu *et al.*, 2022) and is particularly important in experiments where entry of drugs into the brain is at low levels. Due to the high degree of olanzapine entry into the brain, final interpretation of results would not be expected to be affected.
2. Olanzapine metabolites were not included in measurements of overall radioactivity. We did consider this potential problem but having established by LC-MS that the main olanzapine metabolite in rodents, demethyl olanzapine, (DMO; Mattiuz *et al.*, 1997) was not detected in the samples, we concluded that this metabolite did not play a large role in our experiment, likely due to short experimental times (30 min). Another metabolite of olanzapine, 2-hydroxyolanzapine, has been reported to be present in plasma to a similar extent to DMO, although it does not seem to have any pharmacological activity at clinically relevant doses ( Callaghan *et al.*, 1999). Therefore, we assumed that the measurements of radioactivity represent the parent olanzapine.
3. Concentration of the drug injected can be estimated from radioactivity counts by calculating the known amount of added radioactivity per mg of the cold drug in the injectate and refer this calculation to radioactivity in blood samples after 30 min of the experiment. Using this calculation, the concentrations in plasma at the three ages were compared with data obtained using LC-MS. This is illustrated in **Extended data Figure 3** and shows a reasonably good correlation between the two methods reinforcing the validity of using the radioactive compound in our experiments. An additional advantage of using radioactivity is the very high sensitivity of this method allowing measurements to be performed even in very small volumes of samples such as CSF in fetal rats.

### Clinical implications

More than 1200 medications have been prescribed to pregnant and breastfeeding women ( Briggs *et al.*, 2021). As outlined in the Introduction there are little data available from clinical trials upon which clinicians and their patients can make evidenced-based decisions about use of medications during pregnancy and breastfeeding. Even though clinical trials in pregnant women are becoming more accepted ( van der Graaf *et al.*, 2018; Stock and Norman, 2019) it is unlikely that this acceptance will extend to drugs currently in use because of the expense and lack of compensatory profits for the pharmaceutical industry. Our approach is to study drugs used in clinical practice in pregnant and neonatal rats. An important consideration is the extent to which results from studies in rats can be extrapolated to humans. A substantial amount is known about brain development in rats and humans, which allows detailed regional comparisons to be made ( Workman *et al.*, 2013). The placentas in both species are haemochorial; although there are some morphological differences, and are much more similar than many other species that are used for developmental studies ( Ek *et al.*, 2010; Studdert *et al.*, 2020; Møllgård *et al.*, 2017). Of particular importance is the similarity of cellular distribution of ABC efflux transporters in rat and human placenta and brain barriers ( Ek *et al.*, 2010; Saunders & Dziegielewska, 2020; Koehn *et al.*, 2021).

The most striking findings in the present study that may have clinical relevance were the results for transfer of olanzapine across the placenta. Prolonged (longer term) treatment of dams reduced fetal/maternal plasma concentration ratio from 53±10% to 46±6% (p<0.05). If the results are presented as fetal cortex/maternal plasma concentration ratios ( Figure 11) the ratios are not much different from those in Figure 10, indicating that most of the protection of the fetal brain from administration of maternally derived drugs occurs at the placental barrier. If similar measurements can be made in human maternal and umbilical cord blood at birth this would provide confirmation of the protective effect of the drug combinations and would provide some reassurance to both clinicians and their patients that these particular drug combinations would be less likely to harm the baby. In addition, the effect of various drug combinations on olanzapine transfer into maternal brain was quite small, indicating little diminution in the important clinical effect of this therapeutic in the mothers. In contrast, the increase in transfer of olanzapine in the presence of cimetidine and digoxin ( Figure 11) suggests that these combinations should be used with caution, at least until it has been determined if olanzapine does have any deleterious effect on the developing brain. The finding for the combination of olanzapine and paracetamol may be particularly important because of the high rate of self-medication with paracetamol that has been reported in many countries ( Zafeiri *et al.*, 2021).

## Data Availability

Figshare: Underlying data for ‘Entry of the antipsychotic drug, olanzapine, into the developing rat brain in mono- and combination therapies’. https://doi.org/10.26188/c.6273693 ( Huang *et al.*, 2022) The project contains the following underlying data:
• LC-MS raw data • LC-MS raw data.xlsx (Raw data for LC-MS measurements) • LC-MS Skyline template.sky (Skyline template: used to extract peak area from raw data) • Liquid scintillation counting raw data • Olanzapine combination therapy.xlsx (CPM, DPM, weight for all samples as well as sex of all animals) • Olanzapine monotherapy.xlsx (CPM, DPM, weight for all samples as well as sex of all animals) Figshare: Extended data for ‘Entry of the antipsychotic drug, olanzapine, into the developing rat brain in mono- and combination therapies’. https://doi.org/10.26188/c.6273693 ( Huang *et al.*, 2022) This project contains the following extended data:
• Extended data figures.docx (Additional supplementary figures) • Olanzapine Extended data tables.xlsx (Tables of numerical values for each figure) Figshare: ARRIVE checklist for ‘Entry of the antipsychotic drug, olanzapine, into the developing rat brain in mono- and combination therapies’. https://doi.org/10.26188/c.6273693 ( Huang *et al.*, 2022). Data are available under the terms of the Creative Commons Attribution 4.0 International license (CC-BY 4.0).
