# Supplementary material for: Entry of the antipsychotic drug, olanzapine, into the developing rat brain in mono- and combination therapies
Source: F1000Res. 2023 Feb 20;11:1417. Originally published 2022 Dec 2. [Version 2] doi: 10.12688/f1000research.128074.2 (PMC9925881; doi:10.12688/f1000research.128074.2)
Supplement: Peer review history

# Peer review: Reviewer response for version 1

**DOI:** 10.5256/f1000research.140632.r162762

**Published:** 2023-02-13

**Authors:** Udo Schumacher

**Affiliations:** 1 University Medical Center Hamburg-Eppendorf, Hamburg, Germany

The article reports the results of a carefully designed and executed study on the uptake of the antipsychotic drug olanzapine into the developing rat brain. As anti-psychotic drug use is relatively common, this study addresses an important clinical concern about the safe use of these drugs. More importantly, it also addresses the question of drug interaction with other clinically used drugs. These drugs were not randomly chosen as many of them interact with efflux/influx transporters which are also substrates for olanzapine. It would have been helpful to the reader to have been informed about these transporters in the Introduction section and not be introduced to the transport problem in the results section. Maybe even a table showing the interaction of the drugs with specific transporters/pumps may be helpful.

Is the work clearly and accurately presented and does it cite the current literature?

Yes

If applicable, is the statistical analysis and its interpretation appropriate?

Yes

Are all the source data underlying the results available to ensure full reproducibility?

Yes

Is the study design appropriate and is the work technically sound?

Yes

Are the conclusions drawn adequately supported by the results?

Yes

Are sufficient details of methods and analysis provided to allow replication by others?

Yes

Reviewer Expertise:

drug and antibody delivery in malignant tumors and normal tissues.

I confirm that I have read this submission and believe that I have an appropriate level of expertise to confirm that it is of an acceptable scientific standard.

# Response

**Published:** 2023-02-14

**Authors:** Norman Saunders

**Affiliations:** Department of Pharmacology and Therapeutics, University of Melbourne, Australia

On behalf of my co-authors I would like to thank Dr Schumacher for his kind comments about our manuscript. He makes the interesting suggestion that we should include a mention of efflux/influx transporters in the Introduction section and not be introduced to the transport problem in the results section. Maybe even a table showing the interaction of the drugs with specific transporters/pumps may be helpful. This is a complex and poorly understood area of research in the developing brain. When we were drafting the manuscript we considered including such a summary in the Introduction but decided that it would be clearer to readers if in the Introduction we focused on the problem being studied and left possible explanation for findings to the Discussion.

# Peer review: Reviewer response for version 1

**DOI:** 10.5256/f1000research.140632.r157201

**Published:** 2022-12-21

**Authors:** Byron Bitanihirwe

**Affiliations:** 1 Humanitarian and Conflict Response Institute, University of Manchester, Manchester, UK

The present work explores the level of olanzapine transfer across the placenta and its entry into the developing rat brain. The authors focus on three developmental stages and utilise good quality measures (i.e., liquid scintillation counting), which are important strengths of the study. It was found that prolonged exposure to olanzapine can increase its entry into the brains of postnatal animals. Further, combination therapy models that involved the co-administration of lamotrigine and digoxin with olanzapine increased olanzapine transfer into the fetal brain. Finally, placental transfer of olanzapine between the dam and fetus was affected by combination therapy. Overall, the study is informative and provides important evidence that the placental barrier protects the fetal brain from drugs or medication taken during pregnancy.

 Below are some general comments for the authors to consider:

 1)The authors mention the choroid plexus in the keywords, but nowhere in the manuscript is any allusion made to this structure and how it relates to the findings reported in the present study.

 2) Be consistent with the use of min or minutes in the Methods section.

 3) Consider separating the concentration values plotted in Figure 2A for the CSF and Plasma in to two separate figures.

Is the work clearly and accurately presented and does it cite the current literature?

Yes

If applicable, is the statistical analysis and its interpretation appropriate?

Yes

Are all the source data underlying the results available to ensure full reproducibility?

Yes

Is the study design appropriate and is the work technically sound?

Yes

Are the conclusions drawn adequately supported by the results?

Yes

Are sufficient details of methods and analysis provided to allow replication by others?

Yes

Reviewer Expertise:

Translational Psychiatry/Neuropsychopharmacology

I confirm that I have read this submission and believe that I have an appropriate level of expertise to confirm that it is of an acceptable scientific standard.

# Response

**Published:** 2023-02-14

**Authors:** Norman Saunders

**Affiliations:** Department of Pharmacology and Therapeutics, University of Melbourne, Australia

On behalf of my co-authors I should like to thank Dr Bitanihirwe for his positive comments about our manuscript and his suggestions for changes. We have added a brief new paragraph  to the introduction which emphasizes the distinction between entry of drugs across the cerebral blood vessels and via the choroid plexus and CSF. We would therefore prefer to retain choroid plexus as a key word.

 "minutes has been corrected to "min" in the Methods section.

 It is an interesting suggestion that we should display the plasma and CSF data in Figure 2A in separate graphs. However, Figure 2A shows the time course of drug levels following administration and it is informative to be able to directly compare plasma levels that are initially high (as would be expected) with CSF levels in the same animals that are much lower. It also allows direct comparison of the fall in CSF levels which appears slower than the steep fall in plasma levels over time. This is a point being made in the section “Time-course of olanzapine entry into the brain and CSF”  which references this figure.  We would therefore prefer to retain the figure in its present form.
